# Natural product as a lead for impairing mitochondrial respiration in cancer cells

**DOI:** 10.1080/14756366.2025.2465575

**Published:** 2025-02-27

**Authors:** Agnieszka Pyrczak-Felczykowska, Anna-Karina Kaczorowska, Artur Giełdoń, Alicja Braczko, Ryszard T. Smoleński, Jędrzej Antosiewicz, Tristan A. Reekie, Anna Herman-Antosiewicz

**Affiliations:** ^a^ Department of Physiology, Medical University of Gdańsk, Gdańsk, Poland; ^b^ Faculty of Biology, Collection of Plasmids and Microorganisms, University of Gdańsk, Gdańsk, Poland; ^c^ Department of Theoretical Chemistry, Faculty of Chemistry, University of Gdańsk, Gdańsk, Poland; ^d^ Department of Biochemistry, Medical University of Gdańsk, Gdańsk, Poland; ^e^ Department of Bioenergetics and Exercise Physiology, Medical University of Gdańsk, Gdańsk, Poland; ^f^ School of Science, University of New South Wales Canberra, Canberra, Australia; ^g^ Department of Medical Biology and Genetics, Faculty of Biology, University of Gdańsk, Gdańsk, Poland

**Keywords:** Lichens, usnic acid, breast cancer, electron transport chain, complex II

## Abstract

The impact of the isoxazole derivative of usnic acid, **ISOXUS** (formerly known as 2b) on cancer and non-cancerous cell metabolism was investigated. **ISOXUS** significantly reduced the utilisation of most metabolic substrates that produce NADH or FADH2, mitochondrial electron flow and oxygen consumption rate (OCR) in MCF-7 breast cancer cells in contrast to HB2 normal epithelial cells. Molecular docking revealed that **ISOXUS** inhibits mitochondrial respiratory chain complex II, which was confirmed experimentally. Disturbance of electron flow in MCF-7 cells resulted in increased reactive oxygen species (ROS) production. They appeared crucial for **ISOXUS**-induced cancer cell vacuolization and a drop in survival as an antioxidant, α-tocopherol, protected against these processes. These findings indicate that **ISOXUS** is a metabolic inhibitor that targets mitochondrial complex II in breast cancer cells resulting in diminished ATP production and increased ROS formation which translates into reduced cell viability.

## Graphical Abstract

**Figure:**
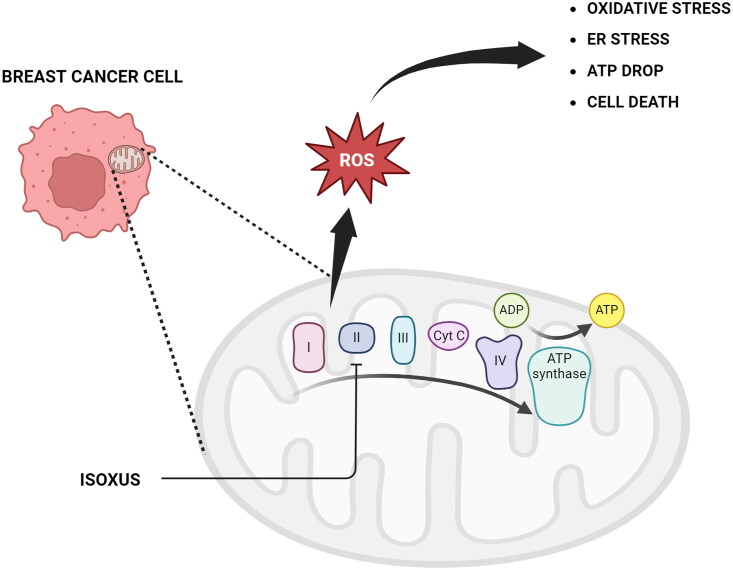

## Introduction

The lichen secondary metabolite usnic acid (UA; C_18_H_16_O_7_) has been implicated in various medically relevant biological activities. These include antibacterial, anti-viral, anti-protozoal, anti-inflammatory, as well as antiproliferative, anti-metastatic and anti-angiogenic activity that might be used in anticancer therapies (reviewed in Ingólfsdóttir, Araújo et al., Luzina and Salakhutdinov, and Solárová et al. ^1–4^). Given this broad action, additional chemical manipulation has been explored to tune its biological activity to a particular target (see Refs.^5–10^ for recent functionalisation studies). This is especially important when exploring the cytotoxicity of UA in both cancerous and healthy cells. UA has displayed cytotoxicity against a broad panel of murine and human cancer cells *in vitro*, albeit in rather high concentrations^11–13^.

In addition to the high concentrations, another obstacle in using UA as an anticancer agent is its toxicity against healthy cells. It revealed hepatotoxic effects when used as a supplement designed for human weight loss^14–16^. Its negative impact on liver cells was observed *in vitro* and *in vivo* (reviewed in Guo et al.^17^). For instance, high levels of serum transaminase and liver necrotic areas were observed in male Swiss mice intraperitoneally injected with UA suspension at a dose of 15 mg/kg/day for 15 days^18^. In 1996, it was already postulated that UA may act as a mitochondrial uncoupler^19^. As a lipophilic weak acid, UA can easily pass the mitochondrial membranes into the matrix, where it releases a proton. The resulting usniate anion diffuses into the intermembrane space to bind to a proton and UA is restored. This cycling causes a proton leak that could dissipate the proton gradient across the membrane, changing the mitochondrial membrane potential. Subsequent reports supported this notion and supplemented it with data showing that UA at higher concentrations also inhibits mitochondrial respiration^20^. In primary cultured murine hepatocytes, 5 µM UA induced necrosis in 98% of cells within 16 h, and it was associated with inhibition and uncoupling of the electron transport chain (ETC) in mitochondria, leading to a reduction in ATP levels in hepatocytes^21^. The analysis of biochemical profiles of rat primary hepatocytes revealed that high doses of (+)-UA (10 or 30 µM) decreased ATP levels. Lowered ATP levels were connected with the depletion of glycogen stores, a drop in glycolysis and TCA, moreover, the mechanism of UA action resembled the action of mitochondrial uncoupler, FCCP^22^. It has also been demonstrated that UA can carry calcium ions across liposomal, mitochondrial, and erythrocyte membranes, thus behaving like a calcium ionophore^23^.

To enhance and tune UA’s activity, we recently reported the synthesis of UA isoxazole derivative **2b**, hereinafter referred to as **ISOXUS**, which was more cytotoxic towards cancer cells than the parental compound while being safer for healthy cells. Moreover, derivative **ISOXUS** induced massive vacuolization of breast cancer cells, but not of normal breast epithelial cells, which resulted from the endoplasmic reticulum (ER) stress and led to paraptosis-like cell death^6^^,^^24^. As ER stress is often connected with mitochondrial function disturbance, this work aimed to elucidate whether the **ISOXUS** derivative impacts cell metabolism and mitochondria in breast cancer cells and to compare the response of cancer and noncancerous cells to this derivative.

## Materials and methods

### Reagents

Culture media (RPMI 1640 and DMEM), supplements (foetal bovine serum, penicillin/streptomycin antibiotic mixture), and trypsin–EDTA solution were purchased from Corning (Corning, NY). Hydrocortisone, DMSO, saponin, thiazolyl blue tetrazolium bromide (MTT), JC-1, DCFDA, oligomycin, and rotenone were from Sigma-Aldrich (St. Louis, MO), and insulin – from Thermo Fisher Scientific (Waltham, MA).

Synthesis of UA derivative, **ISOXUS**, was described previously by us as the synthesis of **2b**^6^. Briefly, a yellow suspension of (+)-UA (5.00 g, 14.50 mmol) in ethanol (45 mL) and pyridine (45 mL) was treated with NH_2_OH·HCl (1.11 g, 15.95 mmol, 1.1 equiv.) and heated at 80 °C for 1.5 h and all solids dissolved. The mixture was cooled to room temperature where a yellow crystalline product was formed. The suspension was diluted with H_2_O (100 mL), then acidified HCl (1 M aqueous), and extracted with EtOAc (3 × 150 mL). The combined organic layers were washed with H_2_O, dried (Na_2_SO_4_), and concentrated under reduced pressure. The residue was purified by flash column chromatography (1:9 ethyl acetate/hexanes) to afford the desired product **ISOXUS** (3.12 g, 63%) as a yellow solid. ^1^H NMR and ^13^C NMR spectra were recorded using a Bruker 300 NMR spectrometer (Billerica, MA) at 300 and 75 MHz, respectively, and match that previously reported (shown in Pyrczak-Felczykowska et al.^6^).

### Cell culture conditions

Human breast adenocarcinoma cell line MCF-7 was from CLS Cell Lines Service GmbH (Eppelheim, Germany). Human mammary epithelial HB2 cells were provided by Dr. R. Sądej from the Intercollegiate Faculty of Biotechnology, University of Gdańsk and Medical University of Gdańsk, Gdańsk, Poland. Cultures of MCF-7 and HB2 cells were maintained in RPMI 1640 or DMEM medium, respectively, supplemented with penicillin–streptomycin mixture and 10% (v/v) FBS. In the case of HB2 cells the medium also contained 5 µg/mL hydrocortisone and 10 µg/mL bovine insulin. Both cell lines were maintained at 37 °C in a humidified atmosphere with 5% CO_2_.

### Bioenergetics

To determine the oxygen consumption rate (OCR) as well as the extracellular acidification rate (ECAR) the Seahorse XFp extracellular flux analyser was used according to the manufacturer’s instructions (Agilent Technologies, Santa Clara, CA). An additional description of the method is described in Skaripa-Koukelli et al.^25^. In brief, cells were seeded (20 000 cells/well in complete DMEM) into a Seahorse cell culture 96-well plate and incubated at 37 °C and 5% CO_2_ overnight. The following day, cells were treated with solvent vector (DMSO) or a 3 μg/mL DMSO solution of **ISOXUS** and incubated for 6 or 24 h. The growth medium was replaced with unbuffered Seahorse XF DMEM before measurement, which had been supplemented with 2 mM glutamine, 10 mM glucose, and 1 mM pyruvate.

The sensor cartridge was hydrated and calibrated using Seahorse XF calibrant (Agilent Technologies, Santa Clara, CA) at 37 °C in a non-CO_2_ incubator overnight. On the day of measurement, the cartridge was taken out of the incubator and the injection ports were filled with 1.5 µM oligomycin (an inhibitor of oxidative phosphorylation), 0.5 µM fluoro-carbonyl cyanide phenylhydrazone (FCCP, a mitochondrial uncoupler), and 0.5 µM rotenone and 0.5 µM antimycin A (inhibitors of complex I and complex III, respectively). Compounds were provided in the Seahorse Cell Mito Stress Test (Agilent Technologies, Santa Clara, CA). Then, the cartridge and the cell plate were put into a Seahorse analyser and the assay was performed. After a brief 3-min mixing, the initial set of measurements was taken under baseline conditions. Following three readings, oligomycin, FCCP, and rotenone/antimycin A were sequentially added to each well. For each compound injection, three readings of both OCR and ECAR were recorded. The protein level in each well was measured by the SRB method. At least three replicates were analysed in each experimental group.

### Mitochondrial functionality assays

Mitochondrial metabolism was assessed by measuring the respiratory chain electron flow using BIOLOG, essentially as described by the manufacturer and in Radogna et al.^26^ Briefly, cells treated with DMSO or **ISOXUS** for 24 h were harvested. The cell suspension was first filtered through a 70-µm filter to remove clumps, and the viability of cells was checked with a trypan blue exclusion test. Cells were suspended in Biolog MAS mixed with Redox Dye MC and saponin (final concentration 35 µg/mL and 90 µg/mL for MCF-7 and HB2 cells, respectively), plated onto MitoPlate™ S-1, which is pre-coated with 31 different substrates of respiration, at a density of 30 000/well, and loaded into an OmniLog Phenotype MicroArray Incubator Reader. The reduction of the tetrazolium redox dye MC, which acts as the final electron acceptor of the respiratory chain, was followed kinetically at 37 °C for 4 h, with 5 min intervals at 590 nm. The initial rate was calculated in the linear phase (first hour, between 5 and 60 min) using the data analysis^®^ 1.7 software (Biolog Hayward, Hayward, CA).

The sensitivity of mitochondria to different inhibitors was determined using MitoPlate I-1 precoated with 22 mitochondrial inhibitors at four dilutions. Cells were plated as described above, except that the assay mix contained succinate as a substrate (4 mM).

### ATP level measurement

Cells were seeded at a density of 1.5 × 10^6^ in a 10 cm diameter plate and allowed to attach overnight. The medium was replaced with a fresh one supplemented with **ISOXUS** (3 µg/mL) or oligomycin (10 µM) for 24 h. The ATP level was measured using the ATPlite Luminescence Assay System based on the production of light caused by the reaction of ATP with added luciferase and d-luciferin (PerkinElmer, Waltham, MA). Briefly, after the treatment, cells were trypsinised and seeded at the density of 4 × 10^3^ per well in a 96-well plate. Cells were lysed for 5 min using the lysis buffer provided in the ATPlite kit. Then, the lysate from treated cells was incubated with the ATPlite substrate for 10 min in the dark. Luminescence was then measured using an EnSpire^®^ Multimode Plate Reader (PerkinElmer, Waltham, MA).

### Molecular docking

AutoDock 4.2.6^27^ was used for the task. Ligand molecules (UA and its derivative) were built using Molden software^28^. The minimised geometry and partial charges were calculated using the PM7 semiempirical method, as implemented in the Mopac program^29^. Protein and ligand files were prepared for docking simulation with AutoDock tools^30^. All possible torsion angles of the computed ligands were able to change. The experimental structure of respiratory complex I – PDB ID 5XTD and complex II – PDB ID 8GS8 were used as a template for modelling. The protein residues directed towards the binding pocket (in complex I: E121, Y204, E208, and E209; in complex II: K92, H99, Y408, E440, and L457) were set as flexible. The centre of the declared docking space was in the centre of the protein binding pocket. The grid spacing was set as 0.375 and 56, 56, 56 and 66, 66, 66 grid points were set in complex I and complex II, respectively. Five hundred Lamarckian genetic algorithm^31^ runs were computed for every calculated system with the following conditions: random position start; 150 individuals in the population; 2 500 000 maximum number of energy evaluations and 50 000 maximum number of generations were used. Three hundred steps of Solis and Wets local search were applied. The lowest energy structure was used for further analysis.

### Complex II activity assay

Cells were seeded at a density of 1 × 10^6^ in a 10 cm diameter plate and allowed to attach overnight. The medium was replaced with a fresh one supplemented with **ISOXUS** (3 µg/mL) or DMSO (control) for 24 h. After the treatment, cells were trypsinised and the complex II activity was measured using the Elabscience^®^ Mitochondrial Complex II Activity Assay Kit which estimates the reduction of 2,6-dichloroindoxol (absorption peak at 600 nm) mediated by Coenzyme Q, the catalytic product of complex II. Briefly, cells were sonicated in extraction solution A at 4 °C and then centrifugated at 600 × *g* for 5 min. The collected precipitate was mixed with extraction solution B containing the inhibitor, sonicated for 1 min at 4 °C, and centrifuged at 15 000 × *g* for 10 min. The supernatant was then collected and the absorbance was measured at 600 nm after 3 s (*A*1) and 3 min (*A*2) using an EnSpire^®^ Multimode Plate Reader (PerkinElmer, Waltham, MA). The mitochondrial complex II activity was calculated as follows:

$$\;\begin{array}{c} \text{Mitochondrial complex II}\\ \text{activity }\left(U/\text{gprot}\right) \end{array}=\frac{\Delta \text{ Asample }\times \text{ Vtotal }\times f\times 1000}{\text{V sample}\times 21.8\times T\times \text{ Cpr }}$$

where ΔAsample is the change OD value of sample (*A*1 − *A*2); *f* is the dilution factor of the sample before the test; Vtotal is the volume of the reaction system, 0.21 mL; Vsample is the volume of the sample, 0.02 mL; 21.8 is the molar absorption coefficient; Cpr is the concentration of protein in sample, gprot/L; *T* is the time of reaction, 3 min.

### ROS and mitochondrial potential measurement

MCF-7 cells were stained with dichlorodihydrofluorescein diacetate (DCFDA), a fluorogenic marker for reactive oxygen species (ROS) in live cells to measure intracellular ROS levels. Mitochondrial membrane potential was evaluated using JC-1 compound. Cells were seeded at a density of 1 × 10^5^ per well at a six-well plate and allowed to attach overnight. The next day, cells were treated with **ISOXUS** or DMSO (control) for 6 or 24 h. Then, the medium was removed and cells were stained with DCFDA (10 µM in PBS) or JC-1 (1 µM in PBS) for 30 min. The fluorescent intensities were quantified using Amnis Flow Sight (Luminex) at excitation/emission maxima of 495/529 nm. Cells were also observed using the Leica DM6000B fluorescence microscope (Wetzlar, Germany).

### Cell viability assays

The impact of antioxidants or aspartate (Asp) on cell viability was determined by the MTT method when α-tocopherol, butylated hydroxytoluene (BHT), or Asp was used or by SRB method when N-acetylcysteine (NAC) was used. Cells were seeded at a density of 4 × 10^3^ per well of a 96-well plate and allowed to attach overnight. The medium was replaced with a fresh one supplemented with the desired concentrations of compounds: 500 μM α-tocopherol, 30 μM BHT, 10 μM NAC, or 10 mM Asp. After 1 h pre-treatment with antioxidants, **ISOXUS** was added for 24 h. In the MTT assay, 25 μL of the MTT solution (4 mg/mL) was added to each well 3 h before the end of the treatment. After incubation, the medium was removed, formazan crystals were dissolved in 100 μL of DMSO and absorbance was measured at 570 nm (with a reference wavelength of 660 nm) in a Victor3 microplate reader. In an SRB method, described previously in Pawlik et al.^32^, 100 μL per well of 10% (w/v) solution of ice-cold trichloroacetic acid was added for 1 h. Plates were washed with water, air dried, and stained with 100 μL of 0.4% sulforhodamine B solution in 1% acetic acid for 15 min. The cells were washed five times with 1% acetic acid and dried. Tris base (10 mM, pH 10.5) was added and the absorbance was measured at 570 nm with a reference filter of 660 nm in a Victor3 microplate reader. Data were obtained from at least three independent experiments performed in triplicate.

### Transcriptome analysis

RNA isolation, library preparation, sequencing, and gene expression profiling have been described in Pyrczak-Felczykowska et al.^24^. Briefly, cells were seeded in 10 cm plates (1 × 10^6^) and treated with 3 μg/mL **ISOXUS** or DMSO for 24 h. Total RNA was isolated using a High Pure RNA Isolation Kit (Roche Diagnostics, Rotkreuz, Switzerland). The library construction and RNA sequencing were performed by the RNA-Seq Service (Macrogen, Seoul, South Korea). Significant differentially expressed genes were analysed for the KEGG pathways (Kyoto Encyclopedia of Genes and Genomes, https://www.genome.jp/kegg/pathway.html, accessed on 25 February 2020). Pathway visualisation was performed using Pathview Web^33^. Data generated during this study are deposited in the NCBI Gene Expression Omnibus (accession number GSE191314; https://www.ncbi.nlm.nih.gov/geo/query/acc.cgi?acc=GSE191314).

### Statistical analysis

The statistical analysis was performed similarly as in our previous works^34^. At least three independent experiments were carried out and data are shown as means ± standard error (SE). Analysis of the data was performed with ANOVA and the post hoc tests indicated in figure legends using GraphPad Prism (version 8, La Jolla, CA). Differences were considered significant at *p* < 0.05.

## Results

### ISOXUS reduces respiration in MCF-7 breast cancer cells more efficiently than in normal cells

**Figure 1.**
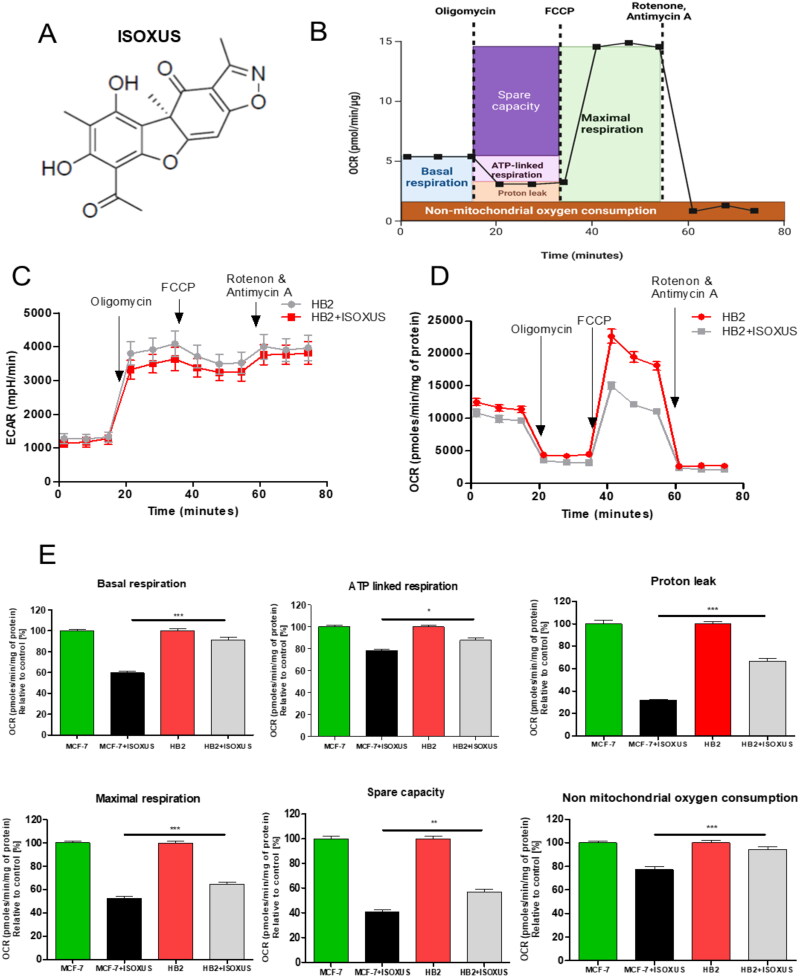
The impact of **ISOXUS** on mitochondrial respiration is less pronounced in HB2 breast epithelial cells than in cancer cells. (A) Chemical structure of **ISOXUS**. (B) Scheme of the Seahorse XFp Cell Mitro Stress Test profile with the key parameters of mitochondrial function. Created using BioRender.com (C) ECAR and (D) OCR of HB2 cells treated with DMSO (controls) or **ISOXUS** (3 µg/mL) for 24 h and analysed by Seahorse. (E) Comparison of different metabolic aspects between HB2 and MCF-7 cells treated with **ISOXUS** for 24 h. The data are shown as the mean ± SE. Statistical significance between **ISOXUS**-treated MCF-7 and HB2 was determined with ANOVA and Tukey’s *post hoc* test and is marked with *p < 0.01, **p < 0.001, and ***p < 0.0001.

It has been shown previously that normal cells are more resistant to **ISOXUS** (structure shown in Figure 1(A)) than cancer cells^6^^,^^24^. To elucidate whether this difference correlates with changes in metabolism, HB2 normal breast epithelial cells were treated with 3 µg/mL **ISOXUS** for 24 h, and the ECAR and OCR were analysed. The principle of the Seahorse XFp Cell Mito Stress Test profile with the key parameters of mitochondrial function is shown in Figure 1(B). **ISOXUS** had a negligible effect on glycolysis of HB2 cells (Figure 1(C)) and influenced only the maximal respiration rate in these cells (Figure 1(D)). Comparison of OCR between MCF-7 cancer cells and HB2 normal cells showed that the reduction of different processes connected with mitochondrial respiration was significantly more pronounced in cancer than noncancerous cells (Figure 1(E)).

### ISOXUS reduces electron flow in the mitochondria of cancer cells

**Figure 2.**
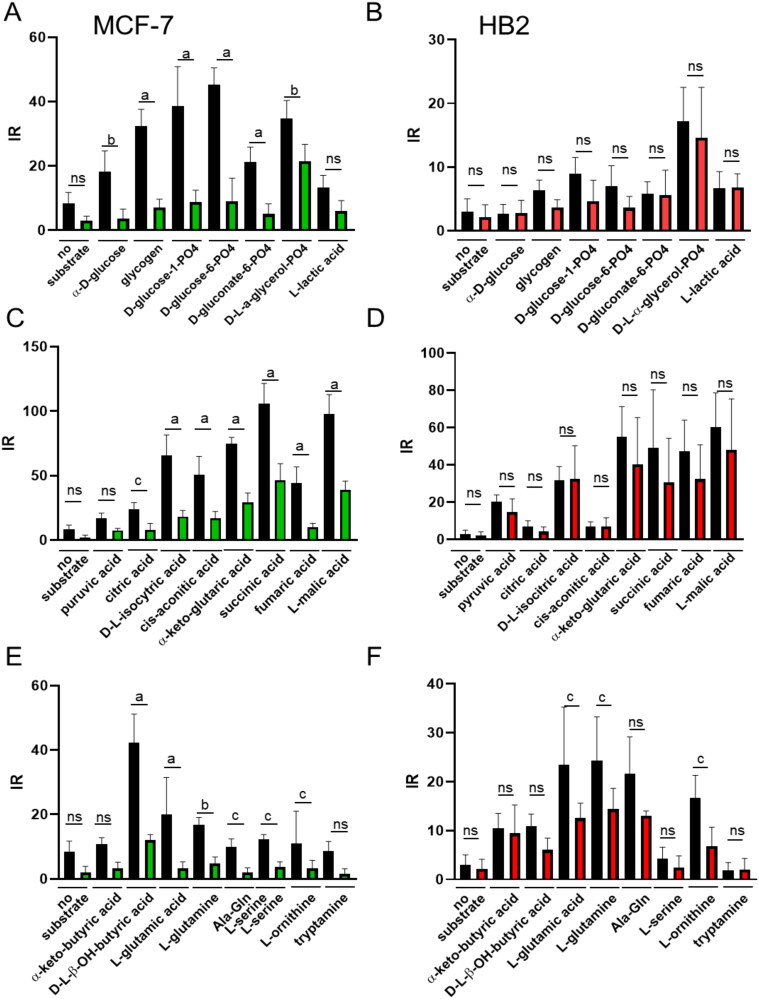
**ISOXUS** blocks the utilisation of metabolic substrates in cancer cells but not in non-cancerous cells. Effect of **ISOXUS** on the utilisation of glycolytic (A, B), Krebs cycle (C, D) substrates or amino acids, lipids and ketones (E, F) in MCF-7 (A, C, and E) or HB2 cells (B, D, and F). Initial rate (IR) values obtained after 1 h of reaction with a particular substrate were calculated using three repetitions and compared to control cells, not exposed to **ISOXUS** and additional substrates. Black columns indicate controls (treated with DMSO), green and red columns indicate samples treated with **ISOXUS**. The data are shown as the mean ± SE. Statistical significance between the non-treated and **ISOXUS**-treated cells was determined with ANOVA and Sidak’s *post hoc* test and is marked with ^a^*p* < 0.0001, ^b^*p* < 0.001, and ^c^*p* < 0.01; ns – non-significant.

Results presented in Figure 1 suggested that **ISOXUS** affects mitochondria mainly in cancer cells. Mitochondrial function can be assessed by measuring the rates of electron flow into and through the ETC from metabolic substrates that produce NADH or FADH_2_. When tetrazolium redox dye acts as a terminal electron acceptor, it is reduced to the purple product which can be detected by the Biolog system. Figure 2 shows the effect of **ISOXUS** on the utilisation of glycolytic (Figure 2(A,B)), Krebs cycle (Figure 2(C,D)) substrates or amino acids, lipids and ketones (Figure 2(E,F)) in MCF-7 or HB2 cells. Initial rate values obtained after 1 h of reaction with a particular substrate were calculated based on three repetitions and compared to control cells not exposed to **ISOXUS** and additional substrates. It can be seen that in cancer cells the addition of the majority of substrates (except some amino acids) increased electron flow; however, treatment with **ISOXUS** reduced this flow significantly (Figure 2(A,C,E)) which indicates downregulation of ETC activity. In contrast, **ISOXUS**-treated HB2 cells saw an insignificant reduction in utilisation of the majority of substrates (Figure 2(B,D,F)).

Analysis of transcriptome in MCF-7 cells using RNAseq technology revealed that **ISOXUS** influenced the expression of genes whose products are involved in glycolysis, TCA cycle, and OXPHOS (Supplementary Figures 1–3). Among downregulated genes were *FBP1*, *PFKM*, *PFKP*, *TPI1*, *GAPDH*, *ENO2*, *PKM*, *LDHA*, *OGDH*, *OGDL*, *SUCLG2*, *ACLY*, *CS*, *Ndufb3*, *COX6B*, and among upregulated genes were *PCK2*, *PGM1*, *HK1/2*, *ACO1*, *IDH1*, *IDH2*, *Ndufa4*, *Ndufb9*, *Ndufs1*, *COX7A*, and *CYC*.

### ISOXUS reduces ATP levels in MCF-7 cells which is connected with reduced respiration

**Figure 3.**
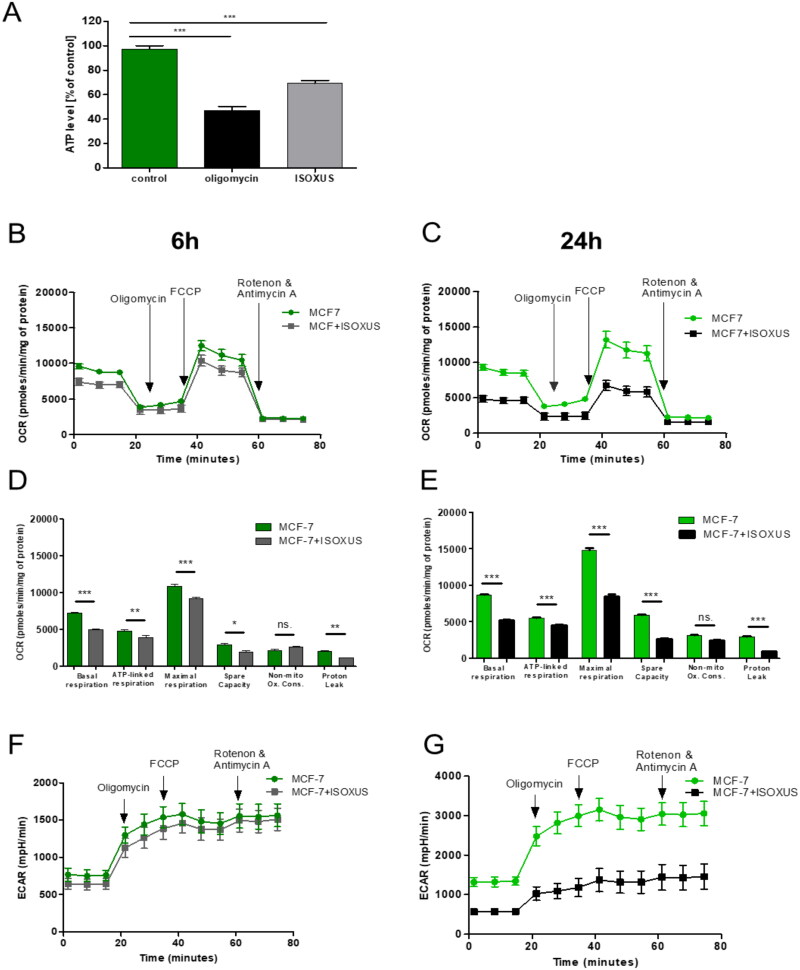
**ISOXUS** decreases ATP levels and disrupts mitochondria functioning in MCF-7 breast cancer cells. (A) MCF-7 cells were treated with DMSO (control), **ISOXUS** (3 µg/mL), or oligomycin (10 µM which blocks mitochondrial complex V) for 24 h. ATP level was measured using the ATPlite Luminescence Assay System (PerkinElmer, Waltham, MA) and was calculated relative to the value for control cells (100%). Statistical significance was determined by ANOVA followed by Tukey’s *post hoc* test: ***p < 0.0001 (B–E) OCR and (F, G) ECAR were measured in MCF-7 cells treated with **ISOXUS** (3 µg/mL) for 6 h (B, D, F) or 24 h (C, E, G). Seahorse tracings and bar graphs compare basal, ATP-linked and maximal respiration, spare capacity, non-mitochondrial oxidative phosphorylation and proton leak between control and **ISOXUS**-treated cells. The data are shown as the mean ± SE. Statistical significance between the respective control and **ISOXUS**-treated fractions was determined with ANOVA followed by Tukey’s *post hoc* test and is marked with *p < 0.01, **p < 0.001, and ***p < 0.0001; ns. – non-significant.

The impact of **ISOXUS** on the level of intracellular ATP was investigated in cells exposed to 3 µg/mL **ISOXUS** for 24 h (at this time the cell cycle arrest was observed^6^). As shown in Figure 3(A), **ISOXUS** reduced ATP levels by 30% compared to control cells. To validate the hypothesis that **ISOXUS** targets cell metabolism, OCR was analysed in MCF-7 breast cancer cells treated with vehicle (DMSO) or **ISOXUS** (3 µg/mL) for 6 or 24 h. As shown in Figure 3(B,C), **ISOXUS** reduced OCR, especially basal and maximal respiration, already after 6-h treatment, although this effect was more pronounced after a longer time. Statistically significant changes were also observed for the ATP-linked respiration, spare capacity and proton leak. Interestingly, **ISOXUS** almost did not affect non-mitochondrial oxygen consumption (Figure 3(D,E)). ECAR, which is an indicator of glycolysis, was reduced only after a longer, 24-h treatment with **ISOXUS** (Figure 3(F,G)).

### Aspartate partially protects against a drop in MCF-7 cell survival caused by ISOXUS

**Figure 4.**
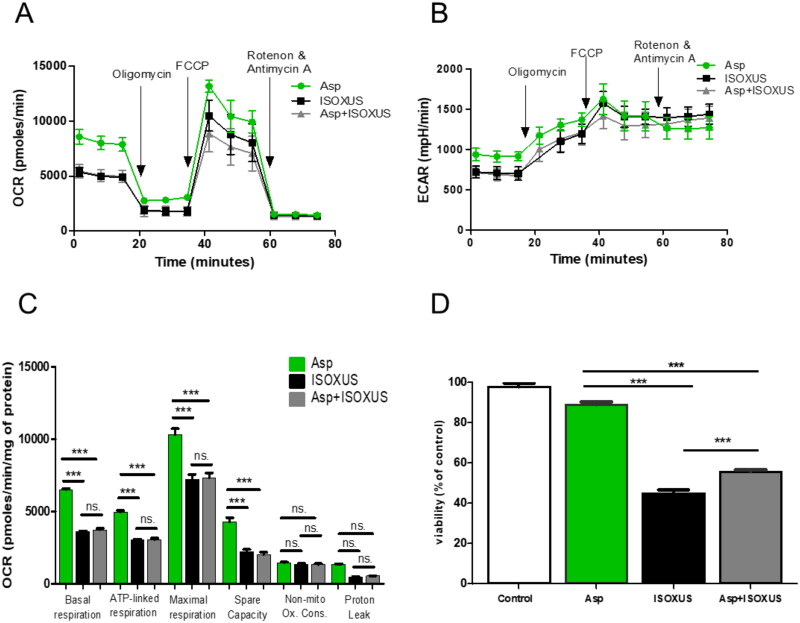
Inhibition of ECT results in aspartate depletion which affects cell viability. Supplementation of MCF-7 culture does not affect the drop in ORC induced by **ISOXUS** (A, C) or ECAR (B); however, it partially protects against **ISOXUS**-induced drop in cell viability (D). Cells were treated with **ISOXUS** (3 µg/mL), aspartate (Asp, 10 mM) or both for 24 h, OCR (A, C) or ECAR (B) was evaluated using Seahorse, and cell viability was measured by MTT test. The data are shown as the mean ± SE. Statistical significance between samples was determined with ANOVA followed by Tukey’s *post hoc* test and is marked with ***p < 0.0001; ns. – non-significant.

ETC inhibition has been shown to decrease intracellular Asp and asparagine levels^35^, and exogenous Asp supplementation to cell culture medium rescued ETC inhibition-mediated proliferation block^36^. To elucidate whether a shortage of Asp is responsible for viability reduction by **ISOXUS**, the treatment effect was compared between cells growing in standard medium and cells cultured in medium supplemented with an additional portion of Asp. As shown in Figure 4(D), Asp partially protected against **ISOXUS**-induced drop in MCF-7 viability. At the same time, it did not affect mitochondrial respiration changes induced by **ISOXUS** (Figure 4(A–C)).

### Impact of ISOXUS on the sensitivity of cancer cells’ mitochondria to inhibitors of ETC complexes

**Figure 5.**
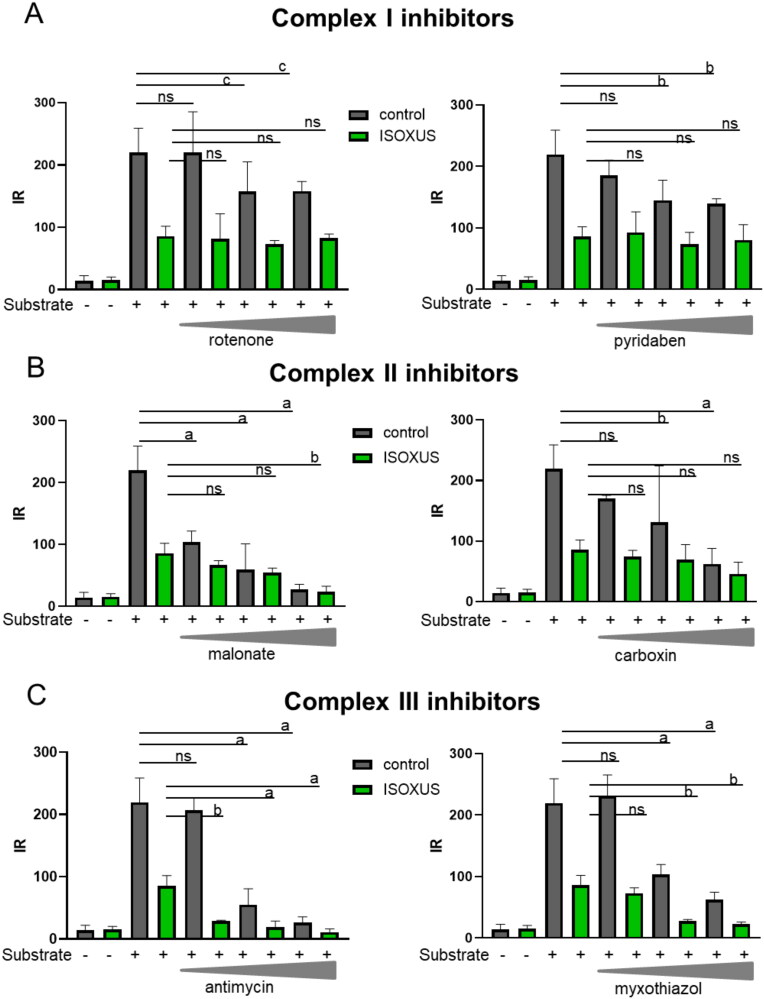
Mitochondrial activity in MCF-7 cells treated or not with **ISOXUS** in the presence of complex I (A), II (B), or III (C) inhibitors and succinate as a substrate. Initial rate (IR) values obtained after 1 h of reaction with a particular substrate were calculated based on three repetitions and compared to control cells not exposed to **ISOXUS** and additional substrates. The data are shown as the mean ± SE. Statistical significance between the non-treated and **ISOXUS**-treated cells was determined with ANOVA and Sidak’s *post hoc* test and is marked with ^a^*p* < 0.0001, ^b^*p* < 0.001, and ^c^*p <* 0.01; ns – non-significant.

Mitochondrial function can be assessed by measuring the sensitivity of mitochondria to diverse inhibitors, including well-known inhibitors of ETC complexes. Using Biolog technology, the activity of **ISOXUS** towards complexes I–III challenged or not with respective inhibitors was evaluated when succinate, which feeds complex II, served as a metabolic substrate. Inhibitors of complex I (rotenone or pyridaben) inhibited electron flow in a dose-dependent manner in control cells. **ISOXUS** decreased electron flow to approximately half of the level in cells treated with anything else besides a substrate; however, it did not potentiate the activity of complex I inhibitors (Figure 5(A)). Similarly, the effect of **ISOXUS** on mitochondrial functioning was almost the same if used alone or with complex II inhibitors (malonate and carboxin) although any of these inhibitors dose-dependently inhibited electron flow (Figure 5(B)). When complex III inhibitors, antimycin or myxothiazol, were used, they inhibited electron flow in a dose-dependent manner and **ISOXUS** potentiated their effects (Figure 5(C)). These results suggest that **ISOXUS** inhibits complex II and possibly complex I activity.

### Molecular docking results

**Figure 6.**
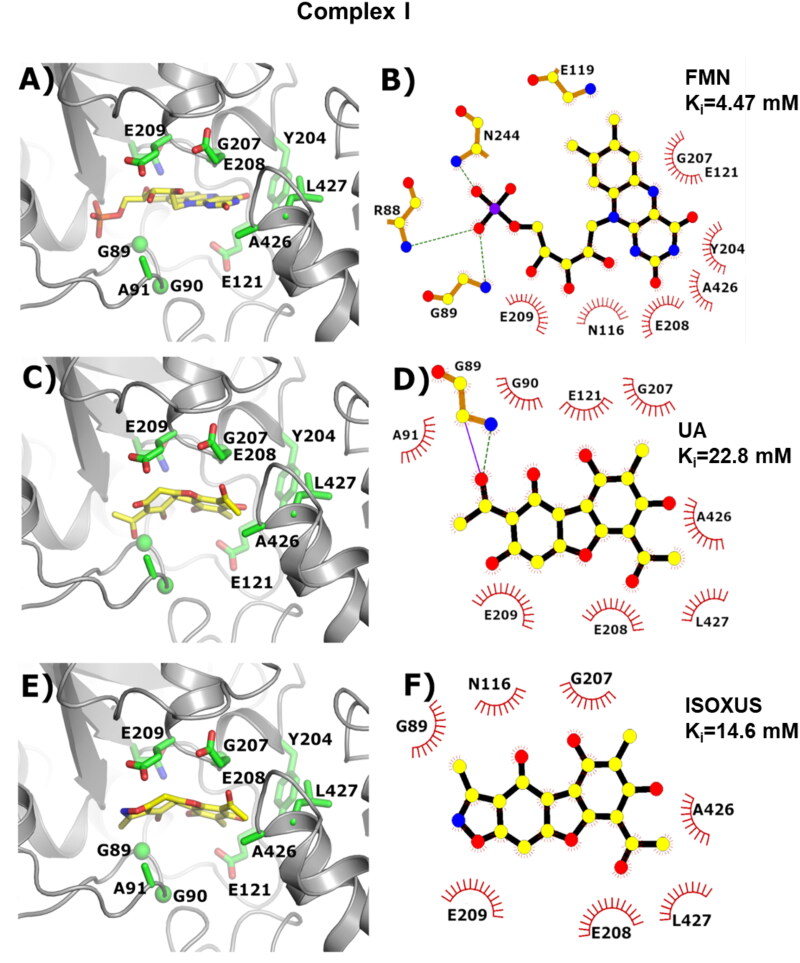
Visualisation of the binding pocket available in the respiratory complex I. On the left side, 3D pictures created by the Pymol software^37^ were given; on the right side, 2D schemes obtained by using ligplot^38^ program were presented. The protein shape was marked grey; the residues involved in the interaction with the ligand were marked green; the ligand was marked yellow. (A, B) The experimental structure (PDB ID 5XTD) of the human respiratory complex I complexed with flavin mononucleotide (FMN). (C, D) Lowest energy model of the UA. (E, F) Lowest energy model of the derivative **ISOXUS**.

**Figure 7.**
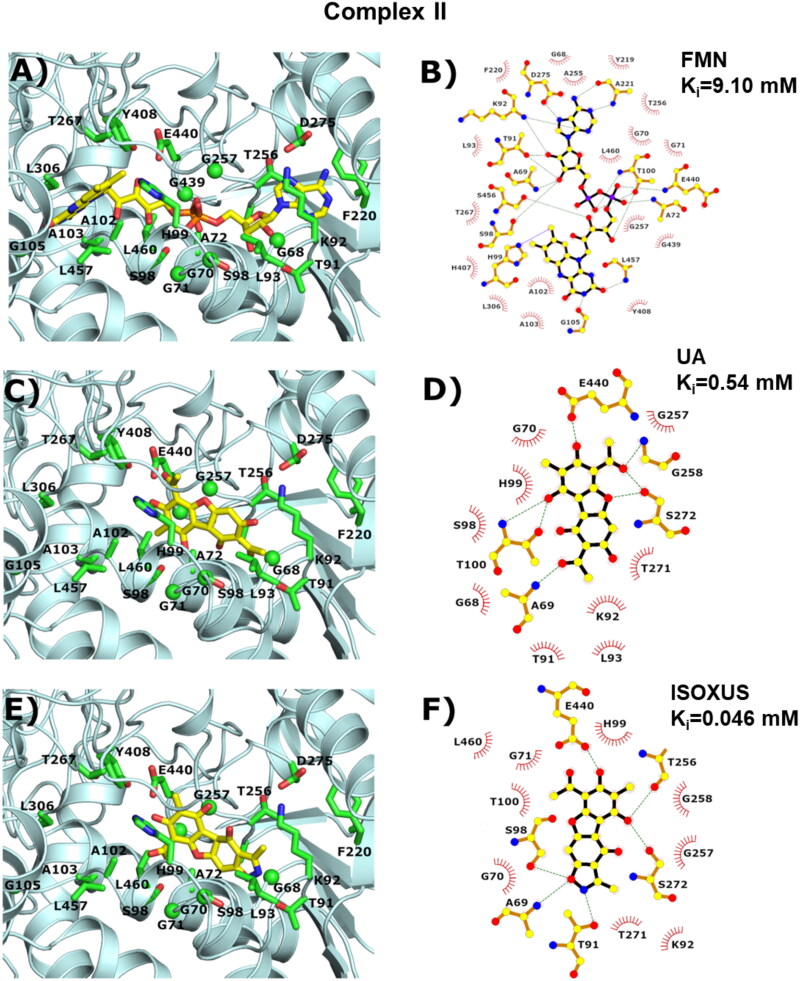
Visualisation of the binding pocket available in the respiratory complex II. On the left side, 3D pictures created by the Pymol software^37^ were given; on the right side, 2D schemes obtained by using ligplot^38^ program were presented. The protein shape was marked cyan; the residues involved in the interaction with the ligand were marked green; the ligand was marked yellow. (A, B) The experimental structure (PDB ID 8GS8) of the human respiratory complex II complexed with flavin adenine dinucleotide (FAD). (C, D) Lowest energy model of the UA. (E, F) Lowest energy model of the derivative **ISOXUS**.

To compare the binding affinity of the UA and derivative **ISOXUS** with complex I and complex II, molecular docking analysis was performed along with flavin adenine dinucleotide (FAD) as the control. The lowest energy structure obtained from the docking simulation and the experimental structures is presented in Figures 6 and 7. The calculated inhibition constants (*K_i_*) for respiratory complex I were 22.8 mM and 14.6 mM for UA and derivative **ISOXUS**, respectively. In comparison, inhibition constants (*K_i_*) calculated for respiratory complex II for the same molecules were as follows: 0.54 mM and 0.0464 mM. The shape of the binding pocket can explain the obtained results. In complex I, the binding pocket is wide. At the entrance to one side, two glutamic acid residues are located (positions 208 and 209). On the other side, two glycine and two alanine residues are present (see Figure 6). The role of pointed glutamic acids is to create hydrogen bond interactions with hydroxyl groups of the ligand. It is clearly visible in the experimental structure (Figure 6(A)). Additionally, two other hydrogen bonds can be created with N116 and Y204. In the case of UA, four additional hydrogen bonds can be created; however, in this case, N116 was replaced by E121. One less hydrogen bond can be created between the protein from complex I and the **ISOXUS**. E209 created the anion–π interaction and the molecule seems to fit better into the binding pocket. Therefore, we observed a very similar inhibition constant in this case.

A completely different situation was observed in the respiratory complex II protein. The ligand molecule (flavin-adenine dinucleotide) is rather big and located in a tight binding pocket. The pocket can be divided into three parts where flavin, linker, and adenine molecules are located. Flavin rings are surrounded mostly by hydrophobic residues, therefore, we did not observe any ligand conformations in this area. At first look, the second pointed area with adenine rings in the centre, seems to be perfect, threonine (T256), and aspartic acid (D275) residue could be a candidate for a hydrogen bond interaction (see Figure 7(A)). However, the UA and its derivative cannot fit here due to steric reasons. As a consequence, only the middle part of the binding pocket was available for the computational search. There are only three residues located in this area capable of creating the hydrogen bond interaction, namely: S98, T100, and E440. And indeed, all of them are involved (see Figure 7(C,E)). However, it does not explain the large difference in the calculated inhibition constant between respiratory complexes I and II. The interaction profiles obtained by the ligplot software pointed to the role of the glycine residues. Since the glycine residues do not have a sidechain, the backbone atoms are available for interactions. A similar situation was observed with the alanine residues since the sidechain is small and directed to the border of the pocket. Together with the flat shape of the binding pocket, it created perfect conditions for low-energy interactions of the protein backbone with UA or derivative **ISOXUS** (see Figure 7(D,F)), which might be the key to the stability of the complex II – inhibitor interaction.

### ISOXUS inhibits complex II activity

**Figure 8.**
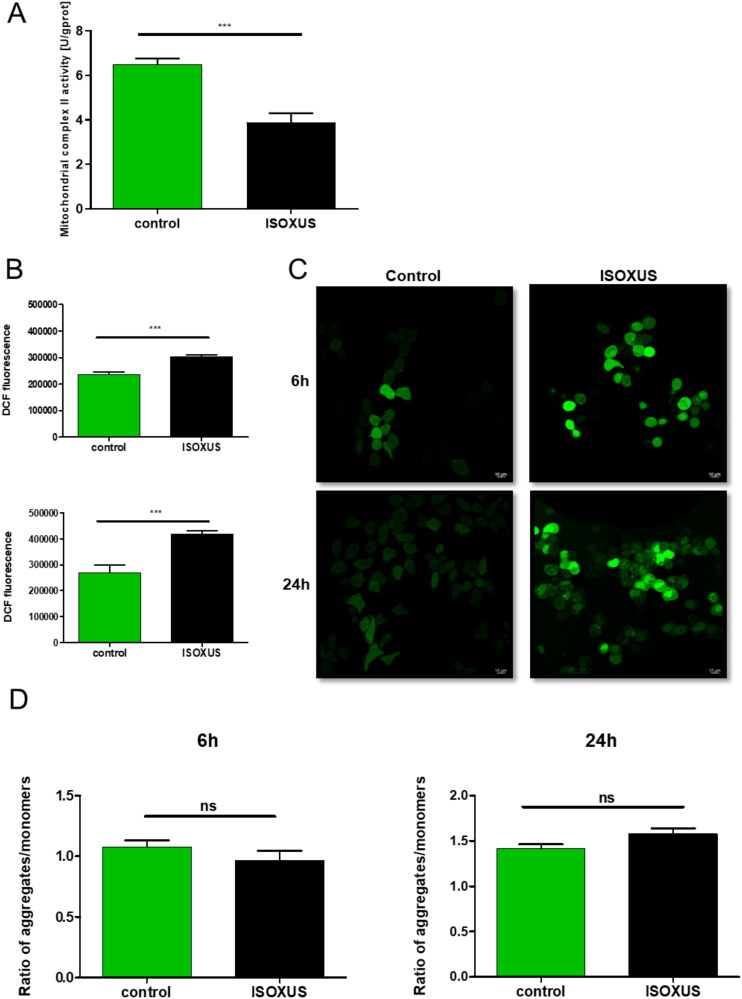
**ISOXUS** inhibits complex II activity and increases ROS formation in MCF-7 cells but has a negligible effect on mitochondrial membrane potential. (A) Cells were treated with **ISOXUS** for 24 h and complex II activity was measured as described in section “Materials and methods”. (B–D) Cells were treated with **ISOXUS** for 6 or 24 h, incubated with 5 µM DCFDA for 30 min or JC-1 (1 µM), and processed for flow cytometry or confocal microscopy analysis of ROS level (B, C) or mitochondrial membrane potential (D). The data are shown as the mean ± SE. Statistical significance between **ISOXUS**-treated MCF-7 and controls was determined with ANOVA, followed by Tukey’s *post hoc* test, and marked with ***p < 0.0001; ns – non-significant.

To experimentally validate molecular docking results, the activity of complex II was measured in cells treated with **ISOXUS** using a commercial mitochondrial complex II activity assay kit and compared to untreated control cells. It measures absorption at 600 nm of the reduced 2,6-dichloroindoxol, which is generated if coenzyme Q is reduced by complex II. Figure 8(A) shows that 24-h treatment with **ISOXUS** reduces complex II activity by 40% compared to untreated cells.

### Mitochondrial dysfunction induced by ISOXUS leads to ROS production

Inhibition of ETC complexes was shown to increase the ROS. To verify whether **ISOXUS** induces oxidative stress, DCFDA was used which, when oxidised, yields fluorescent DCF. As shown in Figure 8, DCF fluorescence increased in **ISOXUS**-treated cells compared with controls, and a statistically significant difference was observed already after 6 h incubation when assessed by flow cytometry (Figure 8(B)) or using fluorescent microscopy (Figure 8(C)). The mitochondrial membrane potential was also investigated; however, results shown in Figure 8(D) demonstrate that it was not significantly affected by **ISOXUS**.

**Figure 9.**
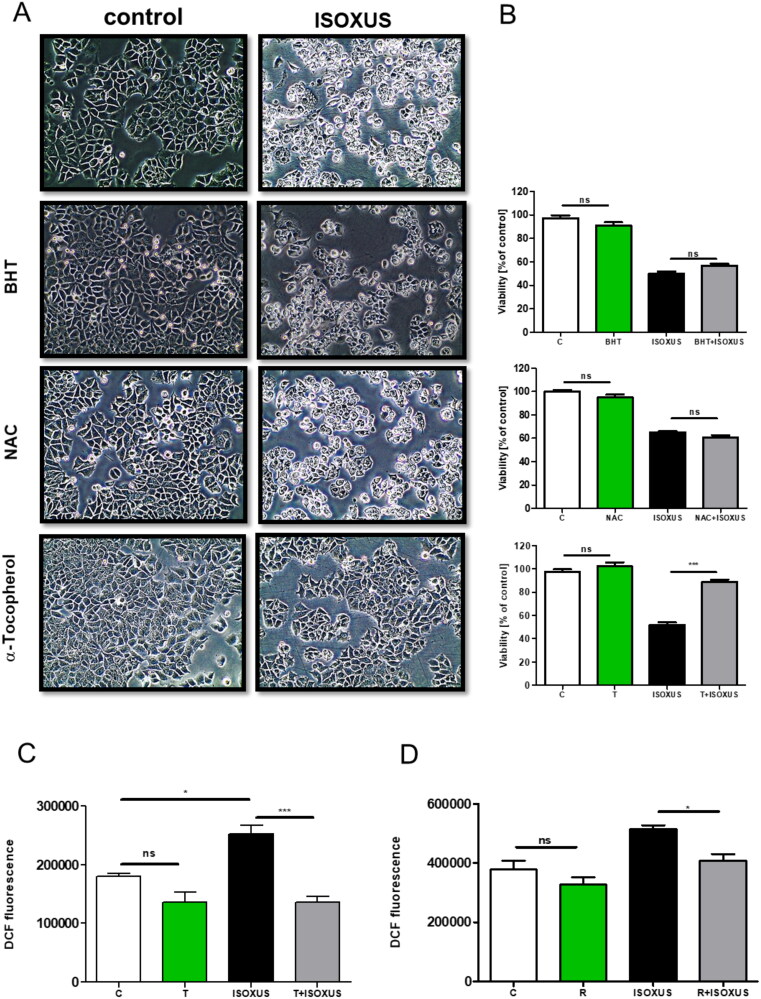
ROS elevated by **ISOXUS** are responsible for cell vacuolization and drop in viability. MCF-7 cells were pre-treated with α-tocopherol (T, 500 µM), butylated hydroxytoluene (BHT, 30 µM), or N-acetylcysteine (NAC, 10 µM) for 1 h and treated or not with **ISOXUS** (3 µg/mL) for 24 h. The morphology of cells was recorded by light microscopy (A) and cell viability by MTT or SRB test (B). (C) Rotenone protects against **ISOXUS**-induced ROS formation. Cells were pre-treated with rotenone (R, 10 µM) for 1 h and treated or not with **ISOXUS** (3 µg/mL) for 6 h. DFF fluorescence was evaluated using a flow cytometer. The data are shown as the mean ± SE. Statistical significance between indicated samples was determined with ANOVA and followed by Tukey’s *post hoc* test and is marked with *p < 0.01, ***p < 0.0001, ns – non-significant.

To elucidate whether the **ISOXUS**-induced drop in cell viability was due to enhanced ROS formation, different antioxidants were applied together with **ISOXUS**. Only α-tocopherol efficiently protected against an **ISOXUS**-induced drop in cell viability as well as vacuolization of MCF-7 cells (Figure 9(A,B)). Increased ROS might be due to the leak of electrons from blocked complex II or complex I when so-called reverse electron transport (RET) occurs. To reconcile these options, rotenone which is a complex I inhibitor was used. Rotenone may stimulate ROS production by complex I when it inhibits forward electron transport or protects against ROS formation during RET. As shown in Figure 9(C), rotenone protected against **ISOXUS**-induced ROS formation which indicates that they were generated by complex I due to RET.

## Discussion

The findings of the present study indicate that **ISOXUS** reduces mitochondrial respiration and electron flow in breast cancer cells in contrast to normal epithelial breast cells. It is supported by the observation that **ISOXUS** decreased OCR already after 6 h exposure while ECAR, which is an indicator of glycolysis, was significantly reduced in MCF-7 cells after 24 h of treatment with **ISOXUS**. Moreover, **ISOXUS** lowered the utilisation of different substrates of cell respiration, even though their surplus stimulated this process in MCF-7 cells non-treated with **ISOXUS**. These changes were less pronounced in noncancerous breast epithelial cells which might explain the selectivity of **ISOXUS** towards cancer cells. Disturbed mitochondrial electron flow led to a drop in ATP level and to enhanced ROS formation which appeared crucial for vacuolization of the cytoplasm (a marker of ER stress) and decreased cell viability.

Generation of ATP relies on a tight coupling of electron transport with oxidative phosphorylation resulting from a proton gradient across the inner mitochondrial membrane. The ETC consists of four complexes (I–IV) and complex V is responsible for the synthesis of ATP. Previous studies searching for mechanisms of hepatotoxicity of a parent compound, UA, indicated that it affected hepatocyte metabolism and/or mitochondria functioning. Abo-Khatwa et al. observed that UA at lower concentrations (below 1 µM) decreased the ADP/O ratio without effect on OCR; however, at concentrations above 1 µM, it inhibited oxygen consumption in isolated mouse liver mitochondria^19^. Protonophoric activities of UA were documented more recently by others using artificial planar bilayer lipid membranes and isolated rat mitochondria^39^. It was shown that UA elevated state 4 respiration in isolated mitochondria (the highest activity at 3–5 µM concentration) and reduced mitochondrial membrane potential^39^. Transcriptomic data seemed to support that UA acts as an uncoupler. Joseph et al. showed that (+)-UA applied at a dose of 200 mg/kg to B6C3F_1_ female mice caused liver mitochondria dysfunctions connected with modulation, mainly upregulation, of expression of genes coding for subunits of complexes I–IV, enzymes of fatty acid oxidations and Krebs cycle^20^. The authors speculated that an increase in the expression of these metabolic genes might be a compensatory mechanism to restore the proton gradient across the inner membrane which is dissipated by UA.

On the other hand, Han et al. showed that 2 µM (+)-UA partially inhibited state 3 respiration and modestly uncoupled mitochondria in primary mouse hepatocytes, while at 5 µM and higher concentrations UA inhibited mitochondrial ETC and reduced ATP level^21^. Uncoupling of mitochondrial respiration was observed in isolated mitochondria in the presence of BSA in a buffer. When BSA was absent, both uncoupling and respiration inhibition were noticed. Moreover, the authors observed increased free radical formation in UA-treated hepatocytes and proved that oxidative stress due to inhibition of the ETC was responsible for the UA-induced death of hepatocytes^21^.

More recently, Sonko et al. using ^13^C labelled glucose demonstrated that UA affected glycolysis and the TCA cycle in isolated rat hepatocytes. They found that UA at 5 or 10 µM concentration significantly reduced cell viability as exposure time increased. Interestingly, the 5 µM UA dose increased oxidative phosphorylation which was interpreted to be an adaptive response to compensate for diminished mitochondrial function; however, both oxidative phosphorylation and gluconeogenesis were dramatically inhibited by 10 µM UA. The authors suggested that UA blocked the TCA cycle downstream from 2-oxo-glutarate, possibly at succinate to fumarate transition which generates FADH_2_ needed by ETC, and it resulted in an ATP level drop^40^.

Our results indicate that **ISOXUS**, the derivative of UA, at 3 µg/mL (8.7 µM) concentration had no significant effect on the mitochondrial membrane potential of MCF-7 breast cancer cells which suggests that uncoupling is not the primary mechanism of action of **ISOXUS**. Rather, the oxygen consumption for proton leak was reduced by **ISOXUS**. The reduction of maximal respiration after FCCP treatment by **ISOXUS** supports the hypothesis that it blocks electron flow rather than acts as an uncoupler. A comparison of the chemical structures of UA and **ISOXUS** also suggests that **ISOXUS** is a weaker acid than UA. Antonenko et al. demonstrated that modification of any of the hydroxyl groups in UA dramatically reduced its uncoupling activity in isolated liver mitochondria and growth-inhibiting activity against *Bacillus subtilis*^39^.

Analysis of the transcriptome of **ISOXUS**-treated cells indicated that this derivative mainly modulates the expression of genes coding for glycolytic and Krebs cycle enzymes, and most of the differentially expressed genes are downregulated. These changes might also impact oxidative phosphorylation and cause a drop in ATP levels, alternatively – they result from the inhibition of ETC.

Our experiments with inhibitors of ETC complexes showed that **ISOXUS**-induced block in electron flow happened at the level of complex I and/or complex II as exposition to respective inhibitors, rotenone or pyridaben inhibiting complex I and malonate or carboxin inhibiting complex II, did not potentiate the inhibitory effect. To reconcile which of the complexes might be a target of **ISOXUS**, a docking analysis was performed. Calculated *K_i_* values for **ISOXUS** with complex II were over 300 times lower than with complex I (0.0464 vs. 14.6 mM). Interestingly, *K_i_* values for UA with complex II were also lower than with complex I (0.54 vs. 22.8 mM) but still 10 times higher than in the case of derivative **ISOXUS** which indicates that the latter is a more potent complex II inhibitor than UA. The shape of the binding pockets can explain such differences. Measurement of complex II activity in control and **ISOXUS**-treated cells confirmed that complex II is a target of this derivative.

Inhibition of ETC complexes is a main source of ROS formation in a cell^41^. Previously, ROS-inducing activity in cancer cells was reported for UA. When used at 12.5–50 µM concentrations, it elevated ROS formation in MCF-7 breast cancer cells and NAC (5 mM) protected against oxidative stress and mitochondria-mediated apoptosis induced by UA^42^. In lung squamous carcinoma cells, UA (10–40 µM) induced oxidative stress which resulted from the inhibition of mitochondrial complexes I and III and reduction of the level of Nrf2, a transcription factor responsible for antioxidant gene expression. Mito-TEMPOL (10 mM), a mitochondria-targeted antioxidant, only partially reversed UA-induced ROS production. Induction of Nrf2 activity by tBHQ (tert-butyl hydroquinone) effectively suppressed UA-induced ROS formation and cell death^11^. In human gastric cancer cells, UA (10–25 µM) increased ROS and NAC protected against DNA damage and cell death induced by UA^43^. On the other hand, necrosis of primary murine hepatocytes treated with UA was blocked only by a combination of vitamin E and BHT^21^. In our model, **ISOXUS** induced oxidative stress in MCF-7 cells which was observed already after 6-h exposure and increased after longer treatment. ROS elevated by **ISOXUS**-induced cell ­vacuolization and decreased viability as the application of α-tocopherol was protective. Interestingly, only this lipophilic antioxidant blocked the antiproliferative activity of **ISOXUS**. It might indicate that the activity of **ISOXUS** is connected with the peroxidation of membrane lipids.

ETC’s main route of ROS production is the leak of electrons from complexes I, II, and III, which reduce oxygen to superoxide^44^^,^^45^. There are two sites of superoxide production at complex I: FMN cofactor which accepts electrons from NADH and the Q binding site where two electrons are transferred to coenzyme Q^46^. Conditions, such as ETC damage or complex I inhibitor, rotenone (blocks Q binding site), stimulate superoxide production by potentiating electron accumulation and FMN reduction. When the Q pool is highly reduced and the proton motive force (Δ*p*, which is the proton concentration and mitochondrial membrane potential) is high, electrons are transported against the redox potential gradient of the ETC and drive back from QH2 to complex I (RET). In such a situation, the Q site of complex I is involved in the production of superoxide which is abolished by rotenone^46^^,^^47^. As we observed the protective effect of rotenone against the **ISOXUS**-induced drop in cell viability, it seems that the primary site of ROS formation is complex I resulting from complex II inhibition.

## Conclusions

This study shows that UA derivative **ISOXUS** inhibits mitochondria functioning at the level of complex II in breast cancer cells. Theoretical results combined with a detailed analysis of the protein binding pocket gave us proof of the selectivity and binding affinity of UA and **ISOXUS** to the analysed respiratory complexes. Inhibition of complex II by **ISOXUS** leads to a drop in oxygen consumption, utilisation of metabolic substrates, and drop in ATP production. It also results in the elevation of ROS, which causes ER stress (cytoplasm vacuolization) and a drop in the viability of breast cancer cells. All these features make **ISOXUS** a promising anti-cancer compound.

## Supplementary Material

Supplementary Figures.docx

## Data Availability

The main text and the Supplementary Material contain all the necessary data to evaluate this study’s conclusions. Additional data generated during the current study are available from the corresponding author upon reasonable request.
