# Supplementary material for: Natural product as a lead for impairing mitochondrial respiration in cancer cells
Source: J Enzyme Inhib Med Chem. 2025 Feb 27;40(1):2465575. doi: 10.1080/14756366.2025.2465575 (PMC11869345; doi:10.1080/14756366.2025.2465575)

**Natural product as a lead for impairing mitochondrial respiration in cancer cells**

Agnieszka Pyrczak-Felczykowska^a^, Anna - Karina Kaczorowska^b^, Artur Giełdoń^c^, Alicja Braczko^d^, Ryszard T. Smoleński^d^, Jędrzej Antosiewicz^e^, Tristan A. Reekie^f^ and Anna Herman-Antosiewicz^g*^

^a^ Department of Physiology, Medical University of Gdańsk, Gdańsk, Poland;

^b^ Collection of Plasmids and Microorganisms, Faculty of Biology, University of Gdańsk, Gdańsk, Poland; ^c^ Department of Theoretical Chemistry, Faculty of Chemistry, University of Gdańsk, Gdańsk, Poland; ^d^ Department of Biochemistry, Medical University of Gdańsk, Gdańsk, Poland; ^e^ Department of Bioenergetics and Exercise Physiology, Medical University of Gdańsk, Gdańsk, Poland; ^f^ School of Science, University of New South Wales Canberra, Canberra, Australian Capital Territory, Australia; ^g^ Department of Medical Biology and Genetics, Faculty of Biology, University of Gdańsk, Gdańsk, Poland

**Supporting Information**

**Figure S1.** Changes in differentially expressed genes of the KEGG pathway related to **glycolysis and gluconeogenesis** after 24-h treatment with **ISOXUS** compared with control cells. Downregulated genes appear in green and upregulated genes appear in red. **(1)** phosphoglucomutase 1; **(2)** hexokinase 1; **(3)** galactose mutarotase; **(4)** fructose-bisphosphatase; **(5)** phosphofructokinase; **(6)** aldolase; **(7)** triosephosphate somerase 1; **(8)** glyceraldehyde-3-phosphate dehydrogenase; **(9)** enolase 2; **(10)** phosphoenolpyruvate carboxykinase 2; **(11)** pyruvate kinase; **(12)** lactate dehydrogenase; **(13)** aldo-keto reductase family 1 member A1; **(14)** aldehyde dehydrogenase 1 family member B1; **(15)** aldehyde dehydrogenase 3; **(16)** acyl-CoA synthetase short chain family member 1.

**
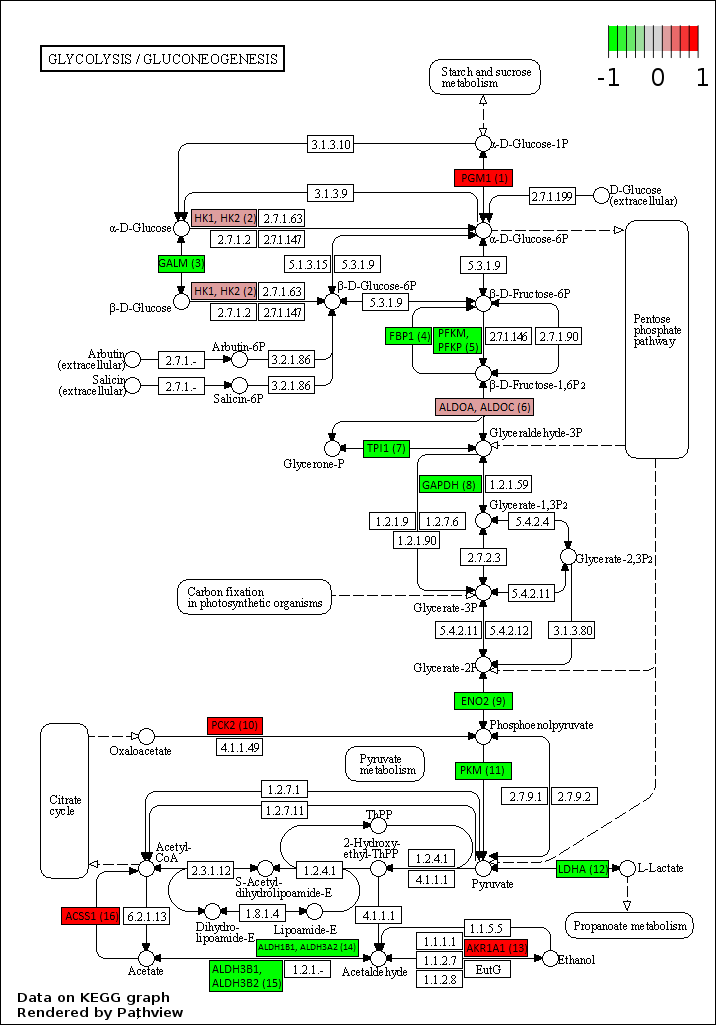
**

**Figure S2.** Changes in differentially expressed genes of the KEGG pathway related to **citrate cycle** after 24-h treatment with **ISOXUS** compared with control cells. Downregulated genes appear in green and upregulated genes appear in red. **(1)** phosphoenolpyruvate carboxykinase 2; **(2)** citrate synthase; **(3)** ATP citrate lyase; **(4)** aconitase 1; **(5)** isocitrate dehydrogenase; **(6)** oxoglutarate dehydrogenase; **(7)** succinate-CoA ligase GDP-forming subunit beta.


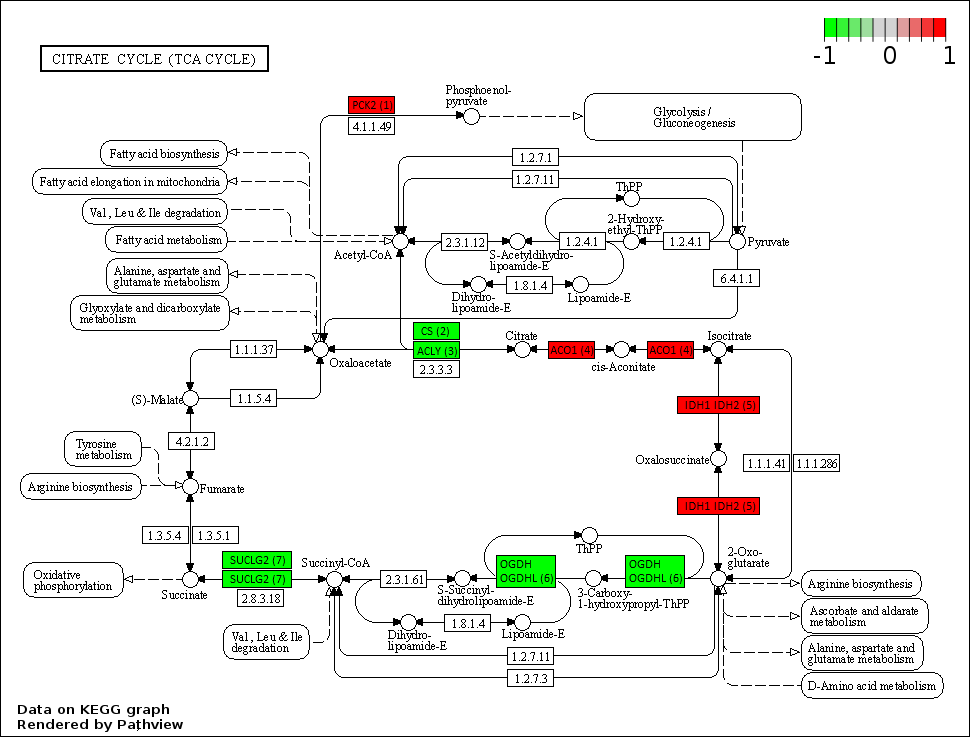


**Figure S3.** Changes in differentially expressed genes of the KEGG pathway related to **oxidative phosphorylation** after 24-h treatment with **ISOXUS** compared with control cells. Downregulated genes appear in green and upregulated genes appear in red.


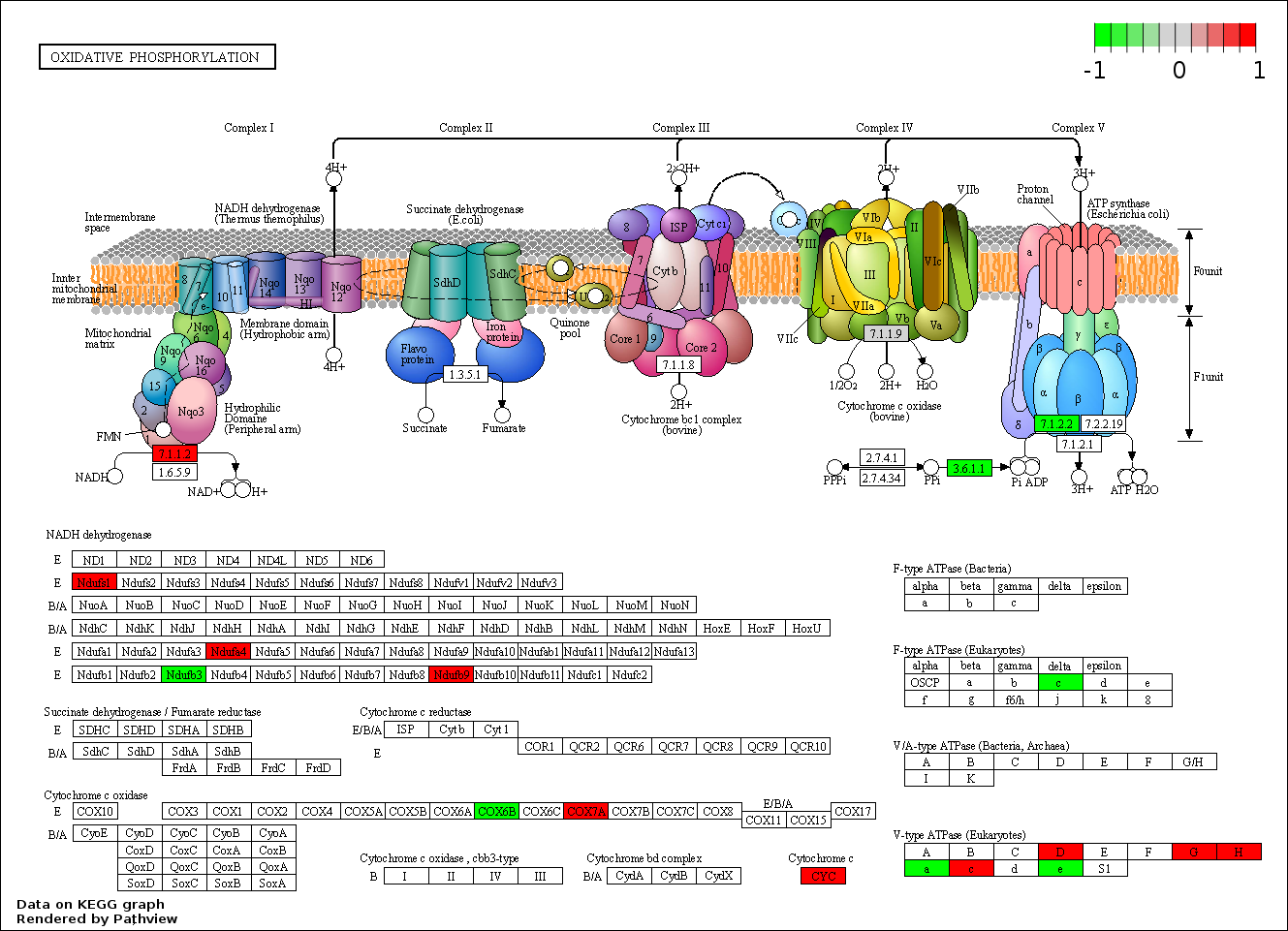

Supplement: Supplementary Figures.docx [file IENZ_A_2465575_SM4961.docx]
